# Established and Emerging Treatments of Skin GvHD

**DOI:** 10.3389/fimmu.2022.838494

**Published:** 2022-02-02

**Authors:** Cornelia S. Link-Rachner, Katja Sockel, Catharina Schuetz

**Affiliations:** ^1^ Medizinische Klinik und Poliklinik I, Medizinische Fakultät Carl Gustav Carus, Technische Universität Dresden, Dresden, Germany; ^2^ Center for Regenerative Therapies Dresden, Technische Universität Dresden, Dresden, Germany; ^3^ Klinik und Poliklinik für Kinder- und Jugendmedizin, Medizinische Fakultät Carl Gustav Carus, Technische Universität Dresden, Dresden, Germany

**Keywords:** Graft-versus-host disease, cutaneous GvHD, acute skin GvHD, chronic skin GvHD, cell-based therapy, targeted therapy, GvHD treatment, GvHD therapies

## Abstract

Graft-versus-host disease (GvHD) of the skin is a severe allo-immune reaction and complication following allogeneic stem cell transplantation. Over the past years, intensive pre-clinical research has led to an improved understanding of the pathophysiology of acute and to a lesser extend chronic GvHD. This has translated into the approval of several new agents for the treatment of both forms of GvHD. This review summarizes the most recent advances in underlying pathomechanisms, clinical trials and newly approved agents for GvHD, with a special focus on skin involvement.

## Introduction

Graft-versus-host disease (GvHD) remains a frequent complication following allogeneic stem cell transplantation (HSCT). Despite better pretransplant conditioning, prophylaxes and graft manipulation, treatment refractory GvHD accounts for 5-30% (1). Skin is the first and most frequently affected organ by GvHD. While local treatment may be sufficient for mild manifestations, systemic immunomodulatory or immunosuppressive therapies are inevitable in moderate and severe forms.

## Cutaneous GvHD: Clinical Presentation

While up to 50% of patients after allogeneic HSCT are affected by any type of GvHD (2), cutaneous lesions are often the first sign of GvHD. A maculopapular rash starting on the décolleté and neck, palms and soles generally spreads to the trunk and extremities as erythroderma. At worst epidermal injury may present itself as bullae which resemble second degree burns or toxic epidermal necrolysis. Itching is one of the first symptoms even before cutaneous lesions occur. Chronic GvHD is more variable in its presentation and may resemble several autoimmune disorders. Cutaneous pathologies include interface dermatitis, lichenoid manifestations, sclerosing dermatitis or panniculitis and fasciitis (3). Sclerodermatous GvHD may evolve toward a scleroderma-like disease with severe functional impairment, some patients becoming wheel-chair bound due to contractures. In addition, chronic cutaneous GvHD is often associated with disturbed wound healing, loss of body hair or alopecia. Advances in the management of skin GvHD are propelled by studies dissecting some of the pathomechanisms underlying GvHD in preclinical models (4).

## Basic Pathomechanisms of Cutaneous GvHD

Pathomechanisms driving GvHD need to be discussed in light of host and donor characteristics. Host predisposing factors refer to the inflammatory milieu at the time of HSCT. A certain degree of skin toxicity, i.e. inflammation or damage, will inevitably follow treatment with chemotherapeutic agents. Upon infusion of donor stem cells, the presence of chronic viral infection and an altered host microbiome may further contribute to a favorable host environment facilitating GvHD.

In acute GvHD, besides T cell mediated inflammation, innate immune cells like neutrophils and monocytes also trigger inflammation through a number of mechanisms including ROS production, release of proinflammatory signals *via* pathogen-associated molecular patterns (PAMP) and danger-associated molecular patterns (DAMP, e.g. ATP, uric acid) released from injured cells (2). DAMPs activate the NLRP3 inflammasome and promote IL-1β-driven cell injury (5). Proinflammatory cytokines and chemokines directly damage epithelial cells, e.g. TNFα or activate donor T cells (6). These migrate to lymphoid tissues where they encounter host antigen-presenting cells (APCs) early after HSCT – as well as emerging donor APCs in the later post-transplant HSCT phase – which will detect antigen histo-incompatibilities (7). So-called “allo”reactive cytotoxic T cells then migrate to the skin causing tissue injury. Of note, various cell types mentioned above may also have regulatory functions preventing or limiting GvHD induced tissue damage.

**Figure 1:**
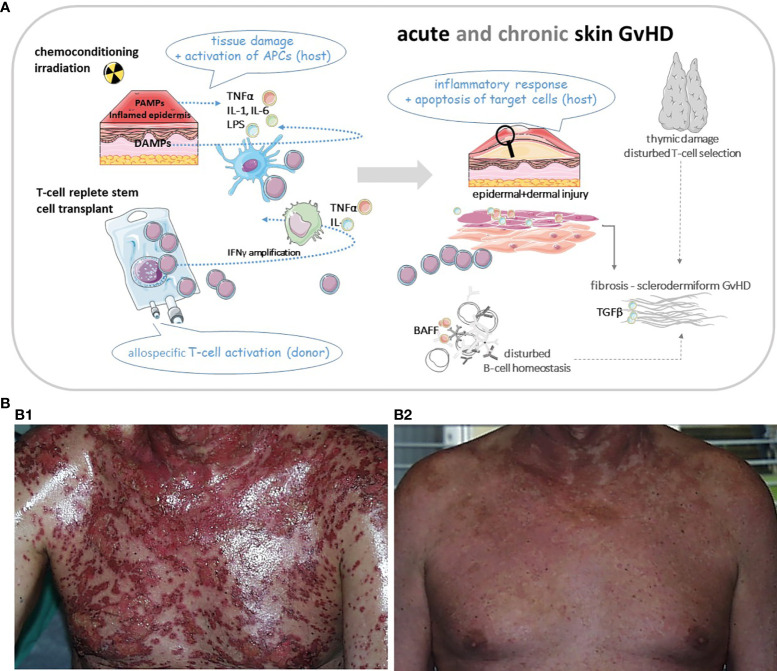
**(A)** Cartoon of basic pathomechanisms in acute and chronic GvHD of the skin. In aGvHD proinflammatory cytokines (TNFα, IL-1 and IL-6) are released from injured tissue following pretransplant conditioning. Increased release of PAMPs (from pathogens within the microbiome) and DAMPs (from damaged cells) drive inflammation through induction of host APCs. These APCs amplify the inflammatory cascade by recruiting cells of the adaptive immune system, e.g. donor T cells and directing T cell migration to lymphoid tissues. Alloreactive T cells differentiate into Th1 and Th17 cells which secrete cytokines like TNFα and INFγ driving alloimmunity. Cytotoxic donor T cells infiltrate the skin or other target organs. In cGvHD cytokines activate the innate and adaptive immune systems or result in direct tissue damage. Thymic injury by alloreactive T cells results in disturbed self-tolerance characterized by decreased regulatory T cells and release of self-reactive T cells. Delayed and dysregulated B cell reconstitution and increased *B cell*–*activating factor* (*BAFF*) levels foster aberrant antibody production to host antigens. Finally aberrant repair of inflamed or damaged skin layers is promoted by activated macrophages, which induce fibroblasts and produce growth factors like TGFβ, resulting in skin fibrosis. APC, antigen presenting cell; BAFF, B cell activating factor; DAMP, damage associated molecular pattern; GvHD, graft-versus-host-disease (aGvHD for acute; cGvHD for chronic); IL, interleukin; INF, interferon; PAMP, pathogen associated molecular pattern; Th, T-helper cell; TNF, tumor necrosis factor. **(B1)** Patient with severe acute skin GvHD (III°). **(B2)** The same patient three weeks after treatment with mesenchymal stem cells. Pictures were provided by Prof. Martin Bornhäuser (MK1, UKD TU Dresden).

While aGvHD is mostly T cell and cytokine driven, the pathophysiology of cGvHD is much more complex. Standardization of nomenclature in order to group different GvH-variants is key to deciphering the following main mechanisms: inflammatory vs. allo/autoimmune-mediated vs. mechanisms resulting in skin fibrosis (8). Moreover, thymic dysregulation leading to poor negative selection, tissue infiltration and injury by Th17 cytotoxic T cells, lack of regulatory T cells and also B cellular hyperactivity with auto/allo-antibody production as well reflect some of the complex mechanisms involved in cGvHD (7) as illustrated in **Figure 1A**.

## Classification, Staging, Grading and Scoring for GvHD

According to the current National Institutes of Health (NIH) classification system, acute GvHD may occur as classic aGvHD within the first 100 days post HSCT, or persistent/recurrent or late-onset aGvHD, reflecting clinical signs of acute GvHD beyond 100 days following HSCT. The latter is typically observed after reduction of immunosuppressive therapy. Chronic GvHD can be divided into the classic form, without signs of aGvHD and the overlap syndrome, combining features of chronic and acute GvHD (9).

In aGvHD the extent or severity of organ involvement falls into 4 stages, and categories are defined for skin, gut and liver (10, 11). For skin involvement, staging reflects the extent of affected body surface area with <25%, 25-50%, >50% and generalized erythroderma plus bullous formation and desquamation >5% from stage 1 to 4. Histopathological grades I-IV describe the epithelial damage ranging from vacuolation of epidermal basal cells to confluent areas of keratinocyte necrosis or even sloughing (12). As for cGvHD, the grading is more detailed with organ specific assessment (13). Notably, so-called “diagnostic” clinical features including poikilodermia, lichen planus, lichen sclerosis and sclerosis are sufficient to establish a clinical diagnosis of cGvHD, while a diagnostic biopsy is mandatory in case of “distinctive” clinical features of cGvHD such as papulosquamous lesions, oral ulcers, onycholysis, or possible alternative diagnoses. **Supplementary Table 1** provides information on how to score severity for cGvHD of the skin.

## Established Treatments of Cutaneous GvHD

While highly desired, there is currently no available tool to predict the occurrence of GvHD safely. A standard GvHD prophylaxis for patients following transplantation consists of a combination of immunosuppressive agents (14). For many years the most common combinations used in Europe consisted of a calcineurin inhibitor such as cyclosporine A in combination with methotrexate. In some institutions cyclosporine A has been replaced by tacrolimus following the finding of 2 phase III trials in which the incidence of grade II-IV acute GvHD was lower with tacrolimus (15). More recently, antithymocyte globulin (ATG) or post-transplant cyclophosphamide have been used as GvHD prophylaxis (16).

### Acute GvHD

The choice of treatment greatly depends on the grade and localization of GvHD. For locally restricted forms of grade I acute skin GvHD, the initial treatment approach will consist of a topical steroid (17). Low-potency topical steroid should be preferred in thinner skin areas (face). Upon grade 2 systemic treatment is required. Standard first-line treatment are systemic corticosteroids such as prednisolone, frequently used at a dosage of 1-2 mg/kg body weight per day (14). A lower dose (< 1 mg/kg) might be sufficient in less severe forms of acute skin GvHD (body surface involvement <50%) and associated with less side effects. Indeed, while being effective in many cases, high dose steroids have numerous adverse effects on metabolism and organs such as bone (18) and steroid-resistance remains an issue that correlates with increasing GvHD severity (19). The treatment of steroid refractory skin GvHD (14) is especially challenging as there are no standardized guidelines available for second-line treatment and trial results of available agents are often limited to single arm studies lacking direct comparability.

A well-established therapeutic option is extracorporeal photopheresis (ECP) (20). Particularly for the treatment of skin-predominant forms of acute GvHD, ECP has been proven to be effective while reducing the cumulative amount of systemic steroids required in clinical trials (12) and real world settings (21). Complete response rates of cutaneous GvHD of >80% have been reported, which also translated into an improved survival and reduction in mortality (12). Importantly, there is increasing data to suggest that an early initiation, preferably within 35 days of onset of aGvHD, achieved an even better response rate (22). In a systemic analysis of 9 studies and >300 patients an overall response rate of 69% was observed across all patients with the highest rate of 84% in cutaneous aGvHD (23). As a limitation this meta-analysis only contained 1 randomized controlled trial indicating the difficulty there is in comparing study results due to a lack of high quality randomized controlled trials. Systemic treatment of acute GvHD may vary depending on local protocols but recent advances in clinical trials and guidelines have aimed at standardizing treatment (17). For localized cutaneous aGvHD manifestations UVA-1 and UVB based phototherapy may be beneficial, resulting in complete response rates of 70% and partial response rates of 24,3% for UVA-1, (24); 57% and 21% for UVB therapy (25). However, treatment response may take weeks to set in.

Antithymocyte globulin (ATG) is also commonly used in patients with steroid-refractory aGvHD. While some older studies have presented data that may indicate a less than expected effect and a higher rate of infections, more recent studies have shown better outcomes [summarized and discussed in (26)]. Recently a study design with a gradual dose escalation scheme has been published with promising outcome, which may be one option in reducing the risk of infectious complications (27). There are no clear guidelines for the second-line therapy of acute skin GvHD in line with the complexity of the disease. Individualized solutions include switching to a different calcineurin inhibitor, (re)introducing mycophenolate mofetil or the mTor inhibitor sirolimus (28).

### Chronic GvHD

The lack of an internationally accepted primary endpoint in clinical trials of cGvHD makes the comparability of previously published trials difficult and the chosen primary endpoints may not automatically translate into a clinical benefit. While this issue has been increasingly addressed in the past years and we are now starting to obtain data from RCTs and discussions on defining a gold standard for the endpoint of these trials (29). Furthermore, in 2014 the NIH re-defined criteria for clinical trials in cGvHD (13).

First-line therapy in the case of a mild cGvHD are topical steroids, mostly higher potency steroids (e.g., clobetasol propionate 0.05%). Cutaneous atrophy or local infections may occur during long-term use of topical steroids; topical calcineurin inhibitors (tacrolimus and pimecrolimus) may spare steroids in some of these patients (30).

Systemic steroids alone or in combination with calcineurin inhibitors are required for severe cGvHD (31). However, the adverse events associated with chronic steroid treatment become particularly apparent in this situation and alternative approaches to spare steroids are especially important. In cGvHD, the use of ECP is one of the best investigated treatment options (29). In a phase 2 trial, ECP resulted in a significantly higher proportion of the subjects achieving partial or complete response of skin manifestations (p< 0.001). A limiting factor was that this assessment was conducted in a non-blinded manner. However, the initial blinded assessment of the total skin score and reduction in steroid use also favored the ECP over the standard care (32).

In a more recent randomized control trial in cGvHD, ECP was added to the standard of care in a first line setting of patients newly diagnosed with moderate or severe cGvHD (33). At 28 weeks, ORR was 74.1% in the ECP arm, compared to 66.7% in the standard of care arm. In addition, quality of life was maintained in the ECP group but declined in the control group.

In an extensive review of 27 studies, where ECP was used in steroid-resistant or dependent cGvHD, the mean response rate for cutaneous cGvHD was 74% (34). A comparable response rate of 64% (ORR) and 27% (CRR) for cutaneous involvement has been previously published in a different meta-analyses (35).

Notably, a recent systematic review of adverse events in second- and third-line treatments for acute and chronic GvHD revealed a lower incidence of infectious adverse events in acute GvHD and lower number of grade 3-5 adverse events in cGvHD in patients on ECP compared to any pharmaceutical management highlighting the safety aspect of this form of treatment (36).

Another therapeutic approach is the use of Rituximab. The anti-CD20 monoclonal antibody is widely used especially in sclerosing cGvHD of the skin, fasciitis, musculoskeletal cGvHD. Clinical responses up to 70% have been described (37). In addition, the tyrosine kinase inhibitor imatinib has been applied for severe, sclerodermic cGVHD (38). Imatinib targets the PDGFR and TGF-β pathways which are involved in skin fibrosis. Besides pharmaceutic interventions, physiotherapy is one of the most useful adjunctive treatments in patients with fasciitis or contractures due to sclerodermiformic GvHD and should always be part of a comprehensive treatment approach (39).

## Emerging Treatments for Cutaneous GvHD

In the past years a number of novel agents have been tested in advanced clinical trials in acute or chronic GvHD and some of these drugs are now available for clinical use. While none of these trials was specifically designed only to assess the effect on skin GvHD, a large percentage of subjects included in these trials were affected by skin GvHD (**Table 1**).

**Table 1:** Emerging treatments for steroid refractory GvHD (sr = steroid refractory).

Drug	Target	sr-aGvHD	sr-cGvHD	Approved	Reference
**Ruxolitinib**	JAK inhibitor	✓	✓	sr-aGvHD sr-cGvHD	(40–42) and (43, 44)
**Itacitinib**	JAK inhibitor	✓			(45)
**IL-2R antibodies**	Interleukin-2 receptor	✓			(46–49)
**TNFa antibodies**	Tumor necrosis factor alpha	✓			(50–54)
**MSCs**	T cell proliferation and T cell response	✓			(55–59)
**Ibrutinib**	Bruton tyrosine kinase (B cells) and interleukin-2-inducible kinase (T cells)		✓	sr-cGvHD	(60)
**Belumosudil**	Rho-associated coiled-coil-containing kinase 2		✓	sr-cGvHD	(61)
**Tregs**	T cell subset with immunosuppressive and immunoregulatory functions		✓		(62, 63)

### Acute GvHD

#### JAK Inhibitors

Ruxolitinib, an inhibitor of the Janus kinase (JAK) 1/2, was approved for the treatment of steroid-refractory acute GvHD in 2019. JAK signaling plays an important role in regulating the underlying immune cells relevant for GvHD such as dendritic cells, macrophages as well as B- and T cells. Most importantly, there is preclinical evidence to suggest that inhibition of the JAK pathway does not negatively impact the GvL effect [as summarized in (40)].

Approval followed a successful open-label, single arm, phase 2 trial (NCT02953678) (41). Of the 70 patients enrolled, 36 had signs of skin GvHD and the overall response rate (ORR) on day 28 was 61% with 25% of patients experiencing a CR. A subsequent phase 3 trial with 309 patients assigned to ruxolitinib (10 mg twice daily) or investigator’s choice of therapy (control arm) was conducted more recently. ORR at day 28 was higher in the ruxolitinib group than in the control group (62% vs. 39%; OR 2.64. 95%CI 1.65-4.22, p<0.001). Overall survival was 11.1 in the ruxolitinib group compared to 6.5 months in the control arm (HR 0.83; 95%CI 0.60-1.15). In this trial the majority (54%) of patients had skin involvement (42). Since ruxolitinib impairs viral specific T cell response, careful monitoring for viral reactivation (including HSV, VZV, CMV) is mandatory. Itacitinib, another JAK1 inhibitor has currently revealed promising results in a phase-1 study on aGvHD, with an overall response rate of about 70% in steroid-refractory aGVH patients (45).

#### IL-2R Antibodies

A number of IL-2R antibodies, namely daclizumab, basiliximab and inolimomab, have been tested in clinical trials for the treatment of aGvHD. These agents generally showed some level of clinical activity in treating aGvHD, especially in cases where skin or gut was involved, (46, 47). However, despite initially promising results, data from subsequent studies were clouded by high rates of infectious complications and in some cases poor long-term survival. In a phase III trial, inolimomab was compared to ATG in patients with steroid-refractory aGvHD (48). The primary endpoint was not met, but long-term follow-up of this study pointed towards a survival benefit of inolimomab (31%) versus ATG [20%, HR 0.572, p=0.03) (49)].

#### Anti-TNFa-Antibodies

The TNFa antibodies infliximab and etanercept have both been investigated for the treatment of aGvHD. Studies with infliximab have delivered mixed results with a modest effect but a generally high rate of infectious complications, (50, 51). There are more studies available for etanercept although the majority of these have yielded a higher response rate than seen in studies with infliximab, but no clear survival benefit has been described (52, 53).

Combining TNFa with IL-2R blocking may be attractive. A prospective trial assessing patients treated with basiliximab in combination with etanercept had an ORR at day 28 of 91% and a two-year OS rate of 55% (54).

#### Mesenchymal Stem Cells (MSC)

A cell-based therapeutic approach is the use of mesenchymal stromal cells (MSCs) in patients with steroid refractory GvHD *(* **Figure 1B**). MSCs have immunomodulatory properties as inhibitors of T cell proliferation and T cell response (55, 56). Several studies show promising results with an overall response on day 28 ranging from 42% to 100% and a complete response ranging from 8%-75% (57). In a prospective study of 69 patients, the ORR was 83% (58). Most recently, a phase III study in pediatric patients was published (59). ORR at day 28 was 70.4% and OS was 74.1% at day 100, with the OR at day 28 being highly predictive of improved survival. An example of a patient with GvHD III° prior and following treatment with MSCs is shown in **Figure 1B**.

### Chronic GvHD

In addition to the well-established first-line treatment with prednisolone and CNIs, several new agents have recently been approved for the treatment of cGvHD.

#### Ibrutinib

In 2017 ibrutinib was approved as a second-line treatment for steroid-resistant cGvHD. Approval came following a successful phase I/II trial (NCT02195869). Forty-two patients with active cGvHD were enrolled who had previously failed 1 to 3 prior treatments. After a median follow-up of 13.9 months the best OR was 67% (60). Notably, analysis by organ domain showed similar responses in the skin (88%) as in the other organs. A phase III trial (NCT02959944) has been completed, but its results have not yet been published.

#### Ruxolitinib

In addition to its positive clinical activity in aGvHD, there are data from a retrospective survey, which indicates that ruxolitinib leads to a high response and 6-month survival rates in heavily pretreated patients afflicted with chronic GvHD (43). Based on these data and the successful trials in aGvHD the prospective, randomized REACH3 trial (NCT03112603) was conducted in 329 steroid-refractory cGVHD patients who received either ruxolitinib or control therapy (44). At 24 weeks OR was 49.7% in the ruxolitinib group (OR 2.99, p<0.001) and failure-free survival was significantly longer (>18.6 months vs. 5.7 months). In this trial approx. 70% of patients had skin involvement, with ~60% having a skin score of greater than or equal to 2. Skin response at week 24 was 41.2% in the ruxolitinib group compared to 15.2% in the control arm. Based on these data the FDA approved ruxolitinib for cGvHD in September 2021 with a recommended starting dose of 10mg given orally twice a day. Studies evaluating the JAK1 Inhibitor itacitinib in the cGVHd setting are currently underway (NCT04200365, NCT035845169). In addition to the systemic use of JAK inhibitors, recent publications point to the benefits of topical application of ruxolitinib by suppressing IFN-γ signaling and T cell infiltration into the skin (64) while sparing systemic side effects. Topical ruxolitinib is currently investigated in clinical trials for cGVHD (NCT03395340, NCT03954236).

#### Belumosudil

Belumosudil is a serine/threonine kinase inhibitor blocking the activity of Rho-associated coiled-coil kinase 2 (ROCK2). The efficacy of belumosudil was evaluated in a phase 2 randomized multicentre trial in cGvHD-patients who had receive 2-5 prior lines of therapy (NCT03640481). Primary endpoint of this study of the best ORR, which was 74% and 77% for belumosudil at 200 mg daily and at 200 mg twice daily, respectively (61). Of note, 38 patients previously treated with ruxolitinib showed a response rate of 68% when treated with belumosudil. In this study 83% of patients had skin involvement and ORR was reported at 37%, with 16% classified as complete responders. Belumosudil was approved by the FDA in 2021 for the treatment of cGvHD after failure of at least two prior lines of systemic treatment.

#### Regulatory T Cells

An innovative cellular approach in the treatment of GvHD is the adoptive transfer of regulatory T cells (Tregs). Tregs are a subset of CD4 T cells with an immunosuppressive and immunoregulatory function (65, 66). The first patients with cGvHD, including skin GvHD, treated with Tregs showed an improvement of GvHD symptoms and systemic immunosuppression could be reduced significantly (62). Adoptive transfer of Tregs was used in a series of five patients with refractory cGvHD (all with a skin GvHD III °). Two of five patients showed improvement of their GvHD symptoms and in four patients immunosuppressive treatment was reduced. Nevertheless, one patient was diagnosed with malignant melanoma and one with Bowen skin cancer several months after the Treg infusion (63).

## Summary and Outlook

Mild acute GvHD of the skin can usually be controlled with standardized treatment protocols. Severe acute skin GvHD requires more aggressive conventional treatments, but newer approaches including cellular therapies are now available. Chronic forms of skin GvHD are the transplant physician’s enemies. Functional impairment and decreased quality of life need to be avoided strenuously. Marked improvement may be obtained with extracorporal photopheresis. Drugs targeting either B cells or the JAK-STAT pathway have opened new treatment perspectives. Regulatory T cells may reverse cGvHD of the skin, but data are still preliminary. It is encouraging that patients who have overcome cGVHD, experience a health status and quality of life similar to those HSCT patients without a history of cGvHD (67).

## Author Contributions

All authors have contributed equally in designing the review article, writing and revising. All authors contributed to the article and approved the submitted version.

## Conflict of Interest

The authors declare that the research was conducted in the absence of any commercial or financial relationships that could be construed as a potential conflict of interest.

## Publisher’s Note

All claims expressed in this article are solely those of the authors and do not necessarily represent those of their affiliated organizations, or those of the publisher, the editors and the reviewers. Any product that may be evaluated in this article, or claim that may be made by its manufacturer, is not guaranteed or endorsed by the publisher.

## Supplementary Material

Supplementary Table 1 Skin scoring of chronic GvHD [adapted from (9)]
