# Supplementary material for: Established and Emerging Treatments of Skin GvHD
Source: Front Immunol. 2022 Feb 2;13:838494. doi: 10.3389/fimmu.2022.838494 (PMC8847139; doi:10.3389/fimmu.2022.838494)
Supplement: Supplementary Table 1 — Skin scoring of chronic GvHD [adapted from (9)] [file Table_1.pdf]

cGVHD severity score:	0	I	II	III
**Skin**<br><u>Score % body surface area (BSA)</u><br>*GVHD features to be scored by BSA*<br>**Check all that apply:**<br>☐ Maculopapular rash/erythema<br>☐ Lichen planus-like features<br>☐ Papulosquamous lesions or ichthyosis<br>☐ Keratosis pilaris-like GVHD		1 - 18% BSA	19 -50% BSA	>50% BSA
**Skin features**<br>Score	☐ No sclerotic features		☐ Superficial sclerotic features "not hidebound" (able to pinch)	**Check all that apply**<br>☐ Deep sclerotic features<br>☐ "Hidebound"<br>☐ (unable to pinch)<br>☐ Impaired mobility<br>☐ Ulceration
**Other skin GVHD features**<br>**Check all that apply:**<br>☐ Hyperpigmentation<br>☐ Hypopigmentation<br>☐ Poikiloderma<br>☐ Severe or generalized pruritus<br>☐ Hair involvement<br>☐ Nail involvement<br>☐ Abnormality present but explained entirely by non-GVHD documented cause (specify):____				
